# Fatigue and the Female Nurse: A Narrative Review of the Current State of Research and Future Directions

**DOI:** 10.1089/whr.2020.0107

**Published:** 2021-03-16

**Authors:** Brennan J. Thompson

**Affiliations:** ^1^ Kinesiology and Health Science Department, Utah State University, Logan, Utah, USA.; ^2^ Movement Research Clinic, Sorenson Legacy Foundation Center for Clinical Excellence, Utah State University, Logan, Utah, USA.

**Keywords:** nursing work, work schedule, patient care, health care, fatigue management

## Abstract

***Background:*** The female nurse exhibits a multitude of personal and environmental characteristics that renders this population especially prone to fatigue. The consequences of fatigue in nurses are widespread and impactful at the personal, organizational, and societal levels. These include high injury rates and burnout in the nurse and poor patient and organizational outcomes.

***Objective:*** This article discusses the implications of fatigue in female nurses, including the impacts of fatigue across multiple entities (*e.g.*, worker, patient, organization). It also reviews the current state of the research, including recent work on nurse fatigue and work schedule characteristics, and key areas for future work that would help empirically establish approaches to counter the detrimental and widespread effects of fatigue.

***Method:*** A narrative literature review was conducted resulting from an analysis of the literature limited to peer-reviewed studies.

***Results:*** A confluence of factors combines to elevate the prevalence and risk of fatigue in the female nurse. Numerous measures have established that performance-based fatigue results from nursing work schedules in nurses. Data also demonstrate that fatigue accumulates across successive shifts. Recent evidence supports the use of objective fatigue measures, including psychomotor reaction time and muscle function-related variables. Current gaps in the literature are delineated in the text.

***Conclusions:*** Strategic and well-designed research studies, as well as recent technological advances in fatigue tracking tools have the potential to help workers, administrators, and organizations develop fatigue management programs that could reduce the heavy burdens of fatigue on a multitude of health, safety, and economical outcomes.

The influences of fatigue and its symptoms are of epidemic proportions. The consequences associated with fatigue apply indiscriminately to individuals of all ages, and across all social, educational, ethnic, racial, and economic demographics.^1,2^ Unsurprisingly, reports have revealed that at least some degree of fatigue is to be found in nearly all of the general or working populations.^3–5^ Since the symptoms of fatigue are presented along a continuum from negligible to severe^6^ the consequences can range from practically unobservable to disastrous, depending on the individual circumstances.^7^

The core factors that appear to contribute to the rampant impact of fatigue are (1) the *high total prevalence* of fatigue symptoms that are manifested in the population at large (*e.g.*, Bultmann et al.^3^ reported that only 2% of a population of 12,095 identified as being completely free of fatigue) and (2) the relatively *high proportion* (∼40%–60%) of adults that exhibit *severe fatigue levels.*^6,8,9^ Although fatigue has been the subject of many reports across a multitude of domains—including physiology, psychology, sports science, military, and so on—relatively little has been reviewed with recent updates regarding physical fatigue in the context of the female nursing worker, and updates for future research directions would be warranted and timely. Given that the female nurse exhibits some unique vulnerabilities to fatigue and its consequences—due to interactions among biological, occupational, and environmental factors—further exploration into this area is of interest for researchers and practitioners.

Thus, the aims of this review were to provide an overview of the implications of fatigue focusing on issues pertinent to the female nurse, a brief update on the current state of the research with a particular focus within the work schedule domain, and to provide future directions for research that could shed more light on areas less explored that remain poorly understood. This review will be focused on hospital-based nurses and aides, since this group accounts for a majority (∼60%) of working nurses and is characterized by unconventional, demanding work schedules (*i.e.*, where 12-hour and rotating shifts are typical).^10^

## Current State

### Implications and impact of fatigue in health care

Fatigue is especially prevalent and consequential in the health care labor sector. Nurse-related fatigue has been defined as a “work-related condition that ranges from acute to chronic in nature and can produce an overwhelming sense of tiredness, decreased energy, and exhaustion resulting in impaired physical functioning, cognitive functioning, or both”^10^ (pg. 488). Despite the widespread and indiscriminating distribution, certain populations are at a particularly high risk for developing and maintaining high fatigue levels. Research shows that populations that are especially at-risk for fatigue include females,^1,6,11,12^ nurses,^1,10,13,14^ and those engaged in demanding work schedules (*e.g.*, long, consecutive shifts; shift work; physically demanding workloads).^10,15–18^ In nursing, these influential risk factors are widely prevalent and substantial considering the fact that ∼90% of nurses are female, and demanding work schedules are a hallmark of the nursing profession. Indeed, fatigue occurrence may be twice as common in females than in males,^1,11^ which is likely a function of both biological influences and environment (*e.g.*, demanding domestic duties outside of work, second jobs, *etc.*).^19–21^ For instance, Alsharari^22^ recently reported that female nurses were at a greater risk for having psychosocial difficulties compared to male nurses, and Sundin et al.^23^ showed that having small children was associated with burnout in nurses, which would most likely have a stronger impact on female compared to male nurses given their more often primary caretaker role with young children. However, despite nursing being a female dominated profession, little progress has been made in mitigating fatigue that may result from the domestic and workplace inequity exhibited by female nursing workers compared to their male counterparts. Thus, the manifestation of fatigue and its negative consequences are exponentially increased with the confluence of these known risk factors. Moreover, other precipitous factors include physical, psychological, social, and emotional stresses, which, when combined, form a highly volatile union of fatigue-inducing factors.^11,24^ Many of these precipitating fatigue factors are prominent characteristics inherent to a large proportion of female nurses.^15,24,25^

Recently, considerable attention has been given to the impact of health care provider (including both physician and nonphysician health care workers) fatigue on patient outcomes. For example, the dangers of health care provider lapses on patient-related consequences are described by the findings of James^26^ who reported that an estimated 400,000 deaths and 2 to 4 million nonlethal serious medical events per year are due to health care provider errors. These statistics are especially alarming considering that such errors are largely preventable given that they have been empirically linked, at least in part, to the workers' fatigue.^16,27–30^ Indeed, the fatigue-impaired health care worker plays a significant contribution with regard to negative patient outcomes,^31^ which includes poor quality of care^31,32^ and increased adverse events such as medication errors due to reduced vigilance and attentional lapses.^27,32–34^ Fatigue is also detrimental to the nurses themselves,^30,35^ which helps explain why nurses experience among the highest nonfatal workplace injury rates of any major job sector.^36,37^ Further, previous findings demonstrate that a lack of attention to the human factor component (*e.g.*, worker readiness for duty) in those who perform patient care tasks directly results in the cascade of poor employee/organizational outcomes such as burnout, a range of caregiver health and safety problems, high turnover rates, staff shortages, and the overall health of the organization (*e.g.*, profitability).^38,39^ Thus, the implications of fatigue are pervasive and ubiquitous given that its influence is uniquely positioned to impact both the workers' and patients' health outcomes—which are key metrics linked to the organization's overall success—because of fatigue's influences on the nursing worker who provides the care.

The consequential burdens associated with fatigue are a function of the relationship between fatigue and impaired human performance across multiple domains.^1,16^ For instance, performing tasks while fatigued results in impaired vigilance, neurobehavioral functioning,^16^ postural balance,^40^ and neuromuscular function.^41,42^ Each of these impairments, either individually or combined, can significantly contribute to fatigue-related injuries or accidents.^30,43^ Moreover, these fatigue-related impairments may pose societal risks outside of the work setting, such as increasing by as much as twofold, the risk of a motor vehicle crash when driving home following a long, fatiguing work shift, especially after a night shift.^44^

The inextricable link between the nurse, patient, and organization^38,45^—combined with the health care sector being the largest and among the fastest growing workforce sectors—compounds the impact of high fatigue levels occurring in the nurse and transmits a complex interaction of negative outcomes exponentially across a multitude of individual, organizational, and societal entities.

Research studies have confirmed that high fatigue levels are found in nurses as reported from a variety of subjective and objective fatigue assessment tools.^12,13,21,40,41,46–49^ These findings have been demonstrated in cross sectional/descriptive designs^12,21,49^ showing a high prevalence of fatigue in nurses. In fact, Raftopoulos et al.^49^ found that 92% and 71% of nurses reported experiencing fatigue sometimes and often or very often, respectively. More recently, experimental or longitudinal designs have been conducted and have more directly confirmed that nursing work is not just associated with fatigue, but is an actual cause of fatigue.^40–42,46,48^

### Measurements

Interestingly, subjective and objective measures of fatigue may not necessarily by correlated.^48^ It is important to note that this lack of association (which has been largely overlooked in the literature) suggests that there may be differences between how one may feel or perceive fatigue and how one actually performs (*i.e.*, tasks) when influenced by fatigue. Much of the research examining fatigue in nurses has conventionally taken the form of subjective questionnaires,^50^ and it has only been more recently that objective measures have been used more principally to investigate the effects of nursing work on fatigue. Our recent work, along with a recent study by Ce et al.,^50^ has used a number of different objective, performance-based measures to help not only identify, but also specifically quantify fatigue resulting from nurses working 12-hour shifts. Results from these studies revealed that 12-hour work shifts induced significant declines in neurobehavioral,^40,42^ balance,^40^ and muscle function^41,42,50^ performance domains. Moreover, deleterious changes induced from nursing work, particularly relating to neurobehavioral function, are also supported by the findings of other investigations.^48,51^ Thus, recent work has successfully used objective measurement tools to identify and quantify fatigue that was shown to be induced from nursing work shifts.

Based on the most up-to-date evidence, it appears that neurobehavioral, postural sway, muscle strength, and explosive strength (of both the lower and upper body) assessments are all objective measures that can effectively be used to capture and quantify work-induced fatigue in nurses. The neurobehavioral measure seems to be a particularly promising assessment tool given that it is both highly sensitive to identify fatigue in nurses,^40,42,48^ and may be well suited for the profession (*i.e.*, vigilance is the essence of nursing).^52^ In this context, studies have assessed the neurobehavioral domain by using the Psychomotor Vigilance Test (PVT) as a means to assess vigilance-based reaction time parameters in nurses. The PVT tool is appealing because it is portable (computer-based), relatively short in duration (≤10 minutes) and thus offers the advantages of being able to be performed on the job (*e.g.*, during breaks) and does not require technical equipment or complicated and time-consuming analyses.

It is, however, unknown whether these aforementioned objective assessments (or the constructs they represent) are strongly related, or whether they are providing unique information. For example, it is possible that the neurobehavioral and muscle function constructs are not representing the same fatigue-originating mechanisms. If that is the case, there may be additive value in using more than one type of fatigue tool (including subjective instruments, where appropriate) to increase the accuracy of tracking and predicting fatigue. Indeed, it is possible that a battery of tests may give a more complete picture of the real fatigue presence and associated risks. However, while research may benefit in gaining greater understanding of fatigue and its mechanisms by examining a multiple instrument fatigue model, such an approach would reduce the practical utility of the tools. Thus, the most effective practical fatigue monitoring model would be the one that can identify or predict fatigue responses with the most accuracy in the simplest way possible (by using only one or two of the most sensitive, field-friendly tests).

More research is needed to establish the objective measures that may be the most complimentary in terms of developing a practical multiple-instrument model such that the instruments/constructs do not share a high degree of commonality and thus provide unique and additive information on fatigue without wasting valuable time and resources. Although the use of both subjective and objective tools may be effective for assessing a nurse's fatigue, the objective assessments more directly engage the actual performance capacities of the worker, and thus, have an advantage of connecting directly to task capabilities while also removing the opportunity for subject-based bias from the assessment. Objectivity is a desirable feature because survey answers can be manipulated to make one look less fatigued, such as in the event there were a perceived benefit to showing lower fatigue and better readiness for duty (*e.g.*, more hours, shifts or overtime = higher compensation, or perhaps could result in more days off in a row). While the use of subjective fatigue assessments can be helpful,^31^ it is herein recommended that these traditional subjective assessments are supplemented with more objective measures of fatigue, including the ones described here, to better inform important determinations of nurses' readiness for duty regarding their fatigue and functional performance status. Strategically incorporating these fatigue-tracking instruments and measures offers promising potential to help organizations and individuals better manage both the occurrences of, and most importantly, the consequences of fatigue in the health care setting.

### Work schedules, recovery, and fatigue tracking

Certain work schedule characteristics are known to increase the likelihood and impact of fatigue. For example, fatigue and its negative effects on both nurse and patient outcomes are more prominent in longer shifts (>12 hours).^17,32,53–56^ Although it has rather well been established that longer (>12 hour) shifts may lead to increased fatigue and its consequences, an area that has been less studied is whether, and to what extent, fatigue may accumulate across multiple, successive shifts. Geiger-Brown et al.^51^ examined sleepiness, fatigue, and neurobehavioral performance parameters over the course of three consecutive day or night shifts in hospital nurses. Although they reported progressively increased sleepiness across the shifts, they showed no changes in the mean reaction time as assessed from the PVT. In contrast, a recent study from our laboratory^42^ showed that neurobehavioral functioning using the PVT instrument was significantly reduced not only after working a single shift but also, perhaps more importantly, was reduced significantly further following the third consecutive shift compared to the scores at the end of the first shift. This incremental decline in performance from the end of the first shift to the end of the third shift indicated an accumulation of fatigue across the three shifts. The reason for these conflicting results is unknown, however, methodological and/or nurse population differences may partially explain the discrepancies. Clearly more work is needed to elucidate the effects of accumulating work shifts on different mental and physical performance parameters and to better isolate the point in the successive shift cycle that fatigue begins to accumulate with each successive shift.

It would also be constructive to investigate how recovery characteristics, such as length of time between shifts^57^ or lifestyle factors (sleep, diet, exercise) may impact the accumulation of fatigue. However, the results of the aforementioned two studies are important in that they do empirically establish that fatigue (in different forms) accumulates across successive work shifts for a number of different subjective and objective measures.^42,51^ Such a finding is alarming because it raises concerns for heightened risks for nurses working a commonly implemented schedule of successive 12-hour shifts in a short time period (known as compressed schedules)—and shows that it is not just longer shifts or rotating shifts that are the cause for concern for inducing substantial fatigue, but that an increasing number of shifts worked with low intershift recovery time^21,58^ is an important risk factor as well. Thus, it is of critical importance to improve the understanding on how each of the different work shift schedule factors (*i.e.*, length, succession, rotation, shift type, recovery) specifically influence fatigue so that schedules can be better manipulated to avoid scenarios where fatigue may become a serious concern for the health and safety of both the nurses and patients. In particular, further exploration is needed to more precisely determine work dose-fatigue associations with regard to how many shifts or cumulative work hours (dose) are required before fatigue begins to accumulate across a multiple shift schedule. Further, it would be useful to determine whether the accumulation trend behaves linearly or if it is more curvilinear (*e.g.*, increases to a point and then levels off without further accumulation) to describe the time course profile and help predict the point where the accumulation of fatigue may begin to exceed tolerable levels.

Addressing fatigue symptom management is of pressing importance for the reasons noted above. Fortunately, the characteristics of fatigue offer a rather encouraging aspect in one regard, namely that it tends to be a reasonably modifiable risk factor.^14^ This suggests that its presence, and, in particular, its consequences may be largely preventable with the prudent application of improved tracking tools and management methods/interventions.^59^ In addition to the aforementioned objective fatigue measures (PVT, muscle function) another promising tool for monitoring fatigue in the field may be found in recent advances in wearable devices with fatigue tracking technologies.

Advances in wearable technologies have allowed researchers to use wearable devices and proprietary algorithms based on physiological-based data, such as activity and sleep patterns, to accurately capture and predict fatigue in other working populations (*e.g.*, railroad, commercial aviation industries). Such a tool would be desirable to be used in health care workers because it overcomes fatigue management barriers by combining objective, biologically derived reliability with field-friendly features. Wearable fatigue instruments would have the potential to be easily implemented, and thus help overcome barriers to widespread adoption, because of its user-friendly, cost effective, and practical characteristics. However, more research is needed to examine the currently available wearable technologies in their capacity to accurately and reliably track fatigue specifically in nurses in the context of their work/environment-specific domain.

The ability to modify fatigue as a risk factor in nurses will require establishing scientifically valid, objective, and field-friendly fatigue monitoring tools as a means to (1) increase the sensitivity, accuracy, and scientific basis for identifying and tracking fatigue in the field (*i.e.*, hospital) and (2) help garner widespread acceptance and ultimately adoption of the best tools and techniques so that they can be readily used by organizations, administrators, and nurses in a collaborative effort to better manage the problems associated with fatigue that have become so routine and pervasive in the health care enterprise.

## Other Unresolved Issues for Future Research Directions

One area in the work schedule domain where the research is severely lacking involves investigating recovery enhancing techniques that may offer the potential to reduce fatigue's negative impact, and, in particular, enhance functional recovery (both in magnitude and timeliness). In addition to the fact that fatigue is common following work shifts, it has also been reported that recovery is poorly achieved in-between shifts,^19,21,51^ leading to a chronic level of fatigue that, in theory, would never be sufficiently overcome in the long-term as long as the fatigue and recovery imbalance cycle persists. Some appealing approaches (other than caffeine), which have been underexplored include the potential of napping, sleep schedule adjustments, assistive devices, task variation, exercise, and nutrition and ergogenic aids to both help mitigate fatigue on the job as well as to help restore function more rapidly by augmenting recovery. For example, Chen et al.^21^ reported that nurses who reported regular exercise exhibited lower acute fatigue and better recovery outcomes versus those who did not engage in regular exercise. Further, Henwood et al.^60^ reported that nurses who self-reported high outside of work (leisure) physical activity levels had improved health and well-being as well as lower sickness absence compared to those who had low-level leisure physical activity.

Research is needed in nurses that implements experimental designs to establish the nature of the benefits of regular exercise in nurses and how this may impact fatigue resistance and recovery outcomes. The most effective types of exercise programming for nurses who are fatigue-prone also warrants investigation, such as strength and/or endurance training, as well as the optimal intensity, volume, and frequency of the training components for maximizing fatigue resilience and minimizing exercise-induced fatigue occurrence, and that can elicit results with the least amount of time commitment. Also, it is presently unknown regarding the role that fitness-related attributes play in relationship to fatigue and recovery outcomes in nurses, which may include aspects such as obesity, muscle strength and endurance, aerobic capacity levels, and so on.

Ergogenic aids may be a potentially effective, but currently underappreciated means to not only counteract the occurrence of fatigue but also to enhance recovery processes. For instance, supplements such as *Rhodiola rosea*^61–63^ and Ashwagandha^64,65^ have been shown to be beneficial for mitigating fatigue in various fatigue-prone populations, but have not been applied to nurses and their work-specific settings. Improved sleep habits have also been recommended to be an important means for reducing fatigue-related risks, which should be implemented by making sleep a higher priority both at the organizational and personal levels.^18^ Finally, a recent review^66^ reported that there was moderate evidence for physical fatigue interventions relating to assistive devices and task variations (however, these were noted in nonnurse working populations) while a multitude of other interventions showed limited evidence of efficacy. The authors^66^ concluded that there was an overall lack of strong evidence for physical fatigue interventions of virtually any kind. Thus, more research of a high quality (randomized controlled trials/longitudinal designs) is warranted in the more promising intervention areas to determine the most effective, practical, and sustainable long-term treatment approaches for fatigue and recovery in nurses.

As the literature is significantly overbalanced with nurse fatigue data using females—a direct reflection of the high proportion of females in the nurse population being studied—a considerable gap exists regarding fatigue and the male nurse. While females are clearly highly prone to fatigue, studying fatigue in the male nurse is also important to better understand their unique needs. There is currently a paucity of data that specifically examines the fatigue characteristics, responses, and consequences in male nurses across the physical, psychosocial, work, and domestic domains. While it is likely that there is a considerable “transfer” of fatigue-related principles from what is known based on the female fatigue literature (*e.g.*, that fatigue impairments increase risks for errors or health hazards is likely not a sex-specific issue), there are sure to be areas where male nurses diverge from females, such as the precise manner in which fatigue may be manifested across the physical, psychosocial, work, and domestic domains and the unique interaction of sex-based attributes across domains. Future work is needed to provide more objective data regarding the specific vulnerabilities that male nurses are prone to, so that pointed interventions can be developed and implemented that best address the male nurse needs.

Also, better information is needed on the “profile” attributes of the nurse who is at higher risk for fatigue. For instance, physical factors (*e.g.*, body composition,^67^ health and/or fitness level, influence of specific diseases, sleep^68^), psychosocial (depression, anxiety, social support networks), and work-related (job autonomy, work patterns/schedules,^68^ ergonomic^68^ factors) should be examined to identify the type of individuals who present as a “high risk,” so that they may be more closely monitored or treated accordingly, such as with a custom-tailored fatigue management program. Finally, research is needed that examines the specific factors that extend routine fatigue into the more severe state known as “burnout.” Such work should consider, for example, the inequity and severity of domestic and workplace duties for those with small children and heavy outside of the workplace responsibilities which may include examining the impact of providing better access to childcare resources for at risk working nurses.

### Awareness, key barriers, and solutions

The many burdens associated with fatigue in nurses have resulted in a push for increased awareness and effective management plans. Recently, many respected professional organizations have put forward position statements and guidelines to draw awareness to health care worker fatigue and its consequences.^69–73^ The American Nurses Association^74^ has also clarified that nurses have an “ethical responsibility and duty to their patients to recognize their level of fatigue before accepting patient assignments.” Collectively, these timely position statements call for action to be taken to “mitigate the risks of fatigue” by highlighting individual and employer actions that could increase awareness, address risks, and prevent adverse outcomes.^21,70,72^ The effect has resulted in experts proposing solutions that incorporate “fatigue management” strategies.^16,21,70,71,75^ Any successful fatigue management strategy must include as a foundation the accurate recognition of fatigue so that fatigue can then be mitigated by appropriate measures (*e.g.*, more sleep, work breaks, less consecutive shifts).^21^ Moreover, a successful fatigue management plan will require the burden of its implementation to be a shared responsibility between the employee and the employer across a multitude of levels (*i.e.*, nurse, nurse managers, and administrators).^10,76^ All of these features combined pose a formidable challenge in overcoming barriers to effective fatigue management. In addition to identifying high fatigue risk individuals, the capacity to predict when fatigue would likely elicit performance deficits is another essential element for enhancing the effectiveness of a fatigue management plan^25^ and a necessity for an accurate countermeasure intervention to be implemented at the most critical time points. These fundamental requirements must be met to have the ability to accurately implement, monitor, and personalize evidence-based fatigue management interventions.

## Conclusion

Fatigue is highly prevalent in female nurses due to individual, environmental, and work-specific factors. Fatigue in the nurse is uniquely positioned to have a disproportionally high impact on a multitude of entities (personal, organizational, societal) leading to potentially widespread catastrophic effects when fatigue is in its most severe form in the nursing worker. The extensive burden is due to the strong interaction among the nurse, their patients, and their environment. Demanding work shift characteristics are commonplace in hospital nurses and this review provided evidence that experimentally links certain work schedule factors to amplified fatigue. In addition to longer work shifts, successive shifts worked over a compressed schedule heightens the risk for accumulating fatigue, but this has often been an underappreciated risk factor in this working population. The nature of nursing work and work schedules, unfortunately, offers the potential for a dangerous interaction among a multitude of impactful risk factors. For instance, it is likely that extra long shifts worked in a compressed work schedule with rotating shifts would create an exponentially harmful scenario for the consequences of fatigue to be most substantial. Because of both the scale and the many risk factors that characterize the profession, more work is needed that unravels the personal-, environmental-, and work-specific features that pose the highest risk as well as the more precise time-course of fatigue and recovery responses across and between work shifts.

Further exploration into these areas may help identify the most damaging personal (fitness for duty, age, *etc.*) and work/organizational (work schedules) factors that could be ideal targets for interventions to manage and ultimately minimize fatigue. Enhancing fitness levels, napping, altering work shift features in favor of recovery, ergogenic aids, and restructuring job duties are potential areas where improvements may be made to better manage fatigue in the female nurse. Objective fatigue measuring tools have been successfully used for tracking fatigue in the female nurse over the course of their work shifts, and their use should become more prominent in fatigue management programs due to their scientific validity, reliability, and reduced potential for subject-based bias. Technological advancements in wearable devices that use biological-based information (activity, sleep) to track fatigue are also a promising area for research and have especially encouraging practical application potential.

It must be recognized across all health care stakeholder levels—including legislators, employers/administrators, managers, staff, and nursing workers—that a good portion of the responsibility to mitigate employee fatigue lies beyond that of the individual nurse (*i.e.*, availability of assistive personnel, shift length, scheduling, work load, *etc.*) and unfortunately, these institutions have generally implemented little support to reduce the magnitude, prevalence, and consequences of fatigue in the high risk nursing worker. Engendering institutional/administrative support for potentially effective fatigue management tools and interventions is critical to widespread success, but administrators first need an empirical framework to be able to justifiably inform their decisions and administrative practices. This review provides a synthesis of recent literature that has empirically identified potentially useful techniques and tools that may help identify and track fatigue, which is a critical step in the fatigue management process.

## Abbreviation Used

PVT: Psychomotor Vigilance Test
